# Incretin receptor agonism during pregnancy: implications for mother and baby

**DOI:** 10.1042/CS20258493

**Published:** 2025-12-08

**Authors:** Laura Dearden, Susan E. Ozanne

**Affiliations:** 1 University of Cambridge Metabolic Research Laboratories, Institute of Metabolic Science, Cambridge, U.K.; 2 MRC Metabolic Diseases Unit, Institute of Metabolic Science, Cambridge, U.K.

**Keywords:** developmental biology, hypothalamus, obesity, pregnancy, type 2 diabetes

## Abstract

Obesity has been described by the WHO as the largest health threat facing mankind. More than 55% of pregnancies in the United Kingdom occur in women who are overweight or living with obesity. Obesity in pregnancy increases the risk of developing gestational diabetes mellitus (GDM), a condition that affects one in seven pregnancies globally and is associated with short- and long-term risks for both mother and baby. Therefore, optimising treatment to effectively treat both obesity and GDM in the perinatal period could have wide-ranging benefits for mother and child. Stabilised analogues of glucagon-like peptide-1 (GLP-1) have revolutionised the treatment of metabolic disease and obesity as they promote weight loss and lower blood glucose. However, the wider action of these analogues, especially in the context of pregnancy, is underexplored. In the United States, the number of young female users of GLP-1 receptor agonists (GLP1RAs), such as Ozempic (semaglutide), increased 659% between 2020 and 2023. Ozempic is not currently licensed for use in pregnancy; however, increased semaglutide use in women of reproductive age has resulted in a rise in ‘Ozempic babies’, when women have unplanned pregnancies while using semaglutide. The potential for GLP1RA use prior to or during pregnancy to limit the transmission of obesity risk between mothers with obesity and their offspring by lowering maternal body weight or correcting maternal glycemia has not been explored. Rodent studies suggest that GLP1RA administration to the dam in pregnancy alters fetal growth, and GLP1RA administration directly to neonates alters development of the hypothalamus. However, recent emerging case reports of human pregnancies where exposure to GLP1RAs has occurred through unplanned pregnancies suggest no harm to the fetus. Given both the potential for GLP1RAs to improve health outcomes in pregnant women with obesity and GDM, and the rapidly rising incidence of fetal exposure, we review the current literature base on the effects of semaglutide use in pregnancy on maternal and offspring health and explore potential broader impacts of use of these agents during the perinatal period based on their known site of action.

## Introduction

**Figure 1:**
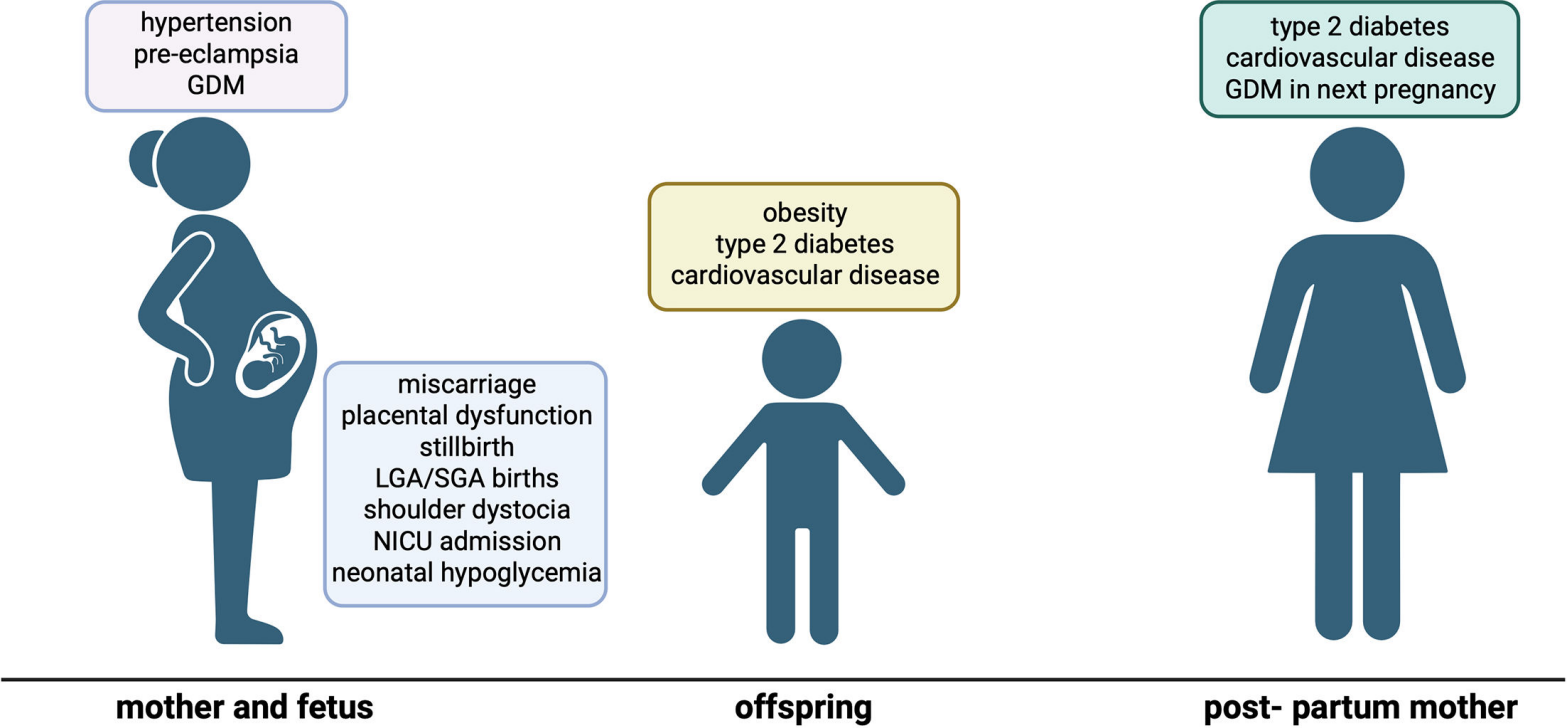
**Consequences of obesity in pregnancy for health of the mother and the baby**. Obesity in pregnancy is associated with immediate health risks for the pregnant mother including hypertension and gestational diabetes (GDM). There is also an increased risk of miscarriage, still birth, shoulder dystocia and NICU admission for the fetus/ newborn. Children born from pregnancies complicated by obesity are more likely to develop obesity, type 2 diabetes and cardiovascular disease themselves as they grow up. There is also evidence that obesity in pregnancy increases the risk of the mother developing type 2 diabetes or cardiovascular disease after the pregnancy and developing GDM in subsequent pregnancies. Improving maternal metabolic health in pregnancy therefore has the potential to improve survival outcomes for the baby, as well as the long-term health of the mother and offspring.

Obesity prevalence has increased exponentially globally. This includes women of child-bearing age; therefore, in many settings, more than half of pregnancies occur in women who are overweight or living with obesity [1]. Maternal obesity has serious implications for the health of both the mother and baby in pregnancy and during birth. Pregnant women with obesity are three times more likely to develop preeclampsia and six times more likely to develop gestational hypertension compared with women who are a healthy weight [2–4], both of which have serious negative consequences and are associated with poor long-term cardiovascular health in both mothers and children (Figure 1). Several large meta-analyses have shown that increasing maternal pre-pregnancy or early pregnancy BMI is associated with increased risks of fetal death, stillbirth, neonatal death and the development of various congenital anomalies [5–7]. Compared with women with a healthy BMI, mothers with obesity have a markedly increased risk of developing gestational diabetes mellitus (GDM) [3,4]. Up to a third of pregnant women with obesity are diagnosed with GDM [8], a global problem affecting one in seven pregnancies. Mothers with GDM are three times more likely to have large for gestational age (LGA) babies [3,9,10], which is in itself a risk factor for complications such as the need for an emergency caesarean section [4,11]. Commencing effective treatment early in pregnancy, before GDM is diagnosed (typically 24–28 weeks gestation) to achieve more rapid normalisation of hyperglycaemia, will therefore have wide-ranging benefits for both mother and fetus [12]. The current first-line treatment for GDM is lifestyle changes such as a change in diet and/or increased physical activity, but in many cases, additional medication is required. Metformin is the first-line pharmacological treatment in many settings and then ultimately insulin in cases when metformin fails to normalise glycaemia. Although effective at maintaining glycemic control, insulin has a number of drawbacks, including need for administration by injection, refrigeration (not feasible in some settings), as well as cost. There is therefore a continued need to explore alternative pharmacological approaches for GDM treatment.

In addition to the immediate negative impact on maternal and fetal/neonatal health, children born from pregnancies complicated by obesity and GDM are more likely to develop obesity and type 2 diabetes in adulthood (Figure 1). This intergenerational transmission of obesity does not simply reflect inheritance of obesogenic genes; human and animal studies demonstrate that the *in utero* environment plays a key role in determining lifelong metabolic health. This likely occurs through changes in fetal development resulting from the altered hormonal and metabolic milieu associated with obesity and GDM in pregnancy. There are currently no treatments for obesity during pregnancy other than lifestyle changes to limit weight gain, and these will often be recommended after the first trimester, as women with obesity tend to present to the clinic later in their pregnancy. This is problematic as there is evidence that maternal BMI at the time of conception is key in determining the transmission of cardiometabolic disease risk, likely due to the fact that several key organ systems involved in cardio-metabolic regulation such as the brain and heart develop early in fetal life.

**Figure 2:**
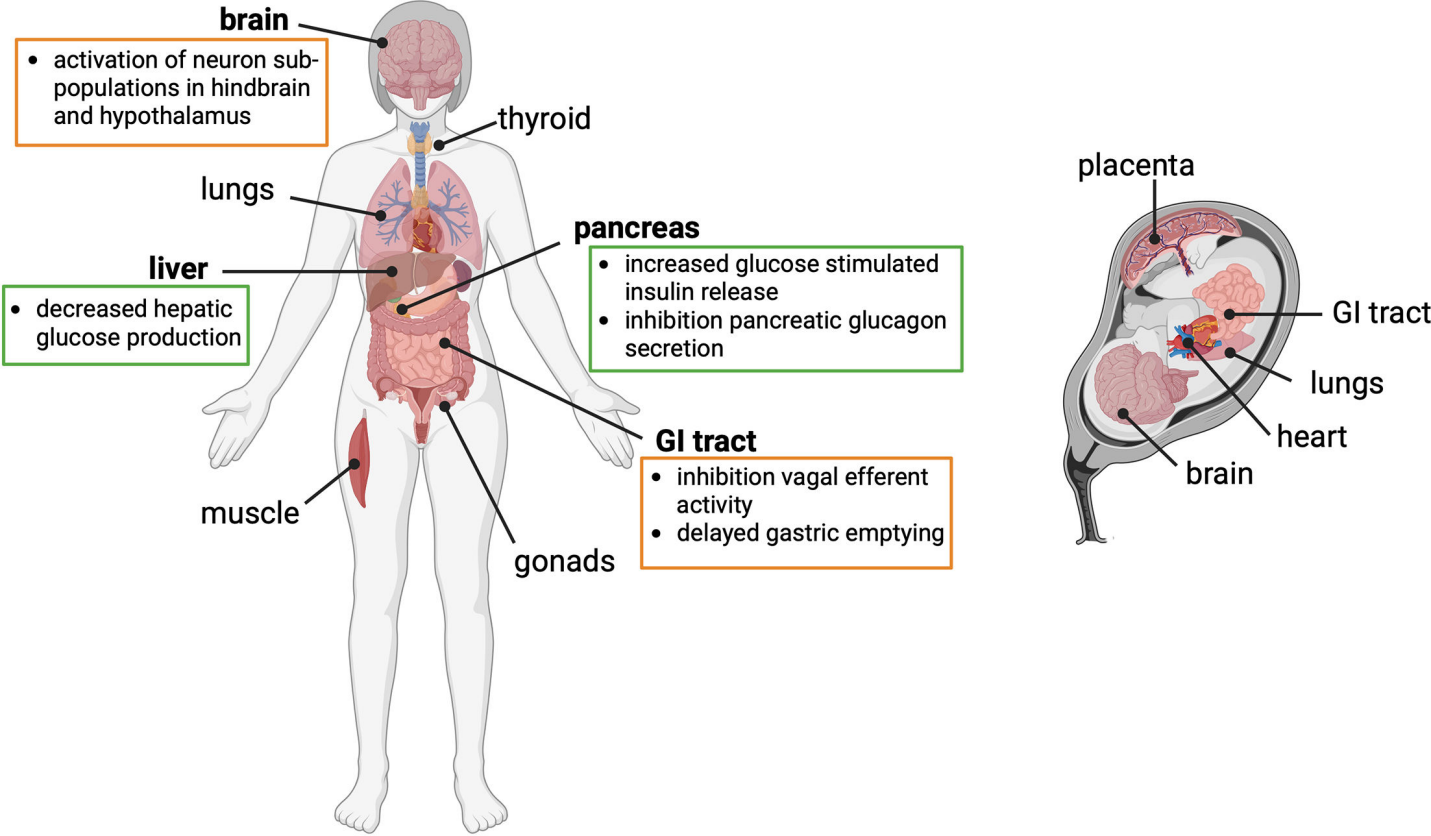
**Confirmed sites of GLP-1 receptor expression in the adult and fetus, and actions of GLP1R activation to lower food intake and glycemia**. Expression of the GLP1R is known to be widespread in adults. The most studied sites of GLP1R expression are the brain and pancreas; however, the receptor is also expressed in other tissues such as the thyroid and gonads. Actions of GLP1R activation which result in reduced food intake shown in orange boxes, actions of GLP1R activation which result in reduced circulating glucose levels shown in green boxes. In the fetus, GLP1R expression has been confirmed in the placenta, brain, heart and GI tract. There is evidence of a rapid increase in GLP1R expression in the lungs at birth. Not only could GLP1R agonists have direct action in the fetus if they cross the placenta, but there also could be increased endogenous GLP-1 release in fetuses in pregnancies complicated by obesity in response to increased circulating fatty acids and glucose. The consequences of activation of GLP1Rs in the fetus are unknown.

In recent years, a major breakthrough has been made in the treatment of obesity in non-pregnant adults with the advent of incretin-based drugs such as semaglutide (marketed as Ozempic and Wegovy), which mimic endogenous glucagon-like peptide 1 (GLP-1) action (see Table 1 for a list of drugs mentioned in this review). GLP-1 is an incretin, meaning it is part of a family of hormones able to stimulate insulin secretion from the pancreatic beta cell in a glucose-dependent manner. It is rapidly released from intestinal L cells in response to macronutrient intake and causes increased pancreatic insulin release, decreased pancreatic glucagon release and reduced gastric emptying (Figure 2). GLP-1 also acts at brain regions involved in the regulation of feeding to lower food intake. GLP-1 receptor (GLP1R) agonists mimic the actions of endogenous GLP-1 to regulate blood glucose levels and reduce food intake [13]. These combined glucoregulatory and satiety-promoting effects ultimately lead to a reduction in body weight in individuals taking the medication. These groundbreaking weight loss drugs will change the lives of countless people who have struggled to maintain a healthy body weight and are proof that drugs, that in part, act through targeting pathways that regulate food intake are an effective route for treating obesity.

**Table 1:** Agonists of the GLP-1 receptor currently available for treatment of obesity or type 2 diabetes that are referred to in this review

Drug name	Action	Structural class	Half life	Approval date by EMA
Dulaglutide	Agonist at GLP1R	Modified human GLP-1	5 days	2014
Exenatide	Agonist at GLP1R	Exendin 4 derivative	4 h	2006
Exendin 4	Agonist at GLP1R	Natural GLP-1R agonist	30 min	N/A
Liraglutide	Agonist at GLP1R	Modified human GLP-1	14 h	2009
Semaglutide	Agonist at GLP1R	Modified human GLP-1	6 days	2018 for T2DM; 2022 for weight loss
Tirzepatide	Agonist at GLP1R and GIPR	Modified human GLP-1 and GIP	5 days	2022 for T2DM; 2023 for weight loss

In the United States, the number of young adult females using GLP-1 receptor agonists (GLP1RAs) increased from 4886 to 37,111 (659.4%) between 2020 and 2023 [14]. The drugs are currently not recommended for use in pregnancy due to a lack of studies in humans. In addition, fetal congenital abnormalities in animal models of GLP1RA use in pregnancy are reported by both Eli Lilly and Novo Nordisk in the prescribing information for Mounjaro (tirzepatide, a dual Glucose-dependent insulinotropic polypeptide [GIP] and GLP1R agonist; Table 1) and Ozempic, respectively. However, due to the ability of the drugs to improve fertility in women with obesity, and their ability to reduce effectiveness of the oral contraceptive pill due to delayed gastric emptying, there are a rapidly increasing number of cases of exposure to GLP1RAs in early pregnancy where women have become pregnant while taking one of the drugs. This led to the UK MHRA releasing an urgent alert in early 2025 reminding women against conceiving while taking the drugs due to the lack of data regarding safety in pregnancy. In the United States, a recent analysis of reports of perinatal exposure to GLP1RAs found a rise from less than 50 cases per 100,000 pregnancies in 2012 to over 200 cases per 100,000 pregnancies in 2020 [15] and a separate study in the US state of Maine showed that perinatal exposure to GLP1RAs rose from 0.02% in 2017 to 0.29% in 2023 [16]. It is concerning that this rapid increase in pregnancy exposure to GLP1RAs is evident even before semaglutide and tirzepatide became more widely available and used for weight loss through private prescription in the United Kingdom and United States.

The ability of GLP1RAs to improve glycaemic control in addition to achieving weight loss means they are potentially a good candidate for improving outcomes in women who have obesity and GDM/diabetes before or during pregnancy, and the long-term health of their offspring. In this review, we will summarise the recent reports of pregnancies where there has been exposure to GLP1RAs and the outcomes for maternal and fetal health after the exposure. We will also examine the studies that have been conducted in animals modelling GLP1RA use in pregnancy and where these give us extra data that cannot currently be collected in a clinical setting. Finally, we will discuss the remaining gaps in our knowledge about the complexities of GLP1RA use in pregnancy and considerations for future studies.

### GLP-1 regulation and function in pregnancy

GLP-1 is an incretin hormone secreted from intestinal L-cells and pancreatic α-cells that augments glucose-stimulated insulin secretion from pancreatic β-cells [17]. In rodents and humans, there is a progressive decrease in circulating GLP-1 across pregnancy [18,19] which, as with insulin resistance that occurs during pregnancy, would cause increased food intake, decreased glucose storage and facilitate shunting of glucose to the growing fetus. In pregnancy, there is a weak association between circulating GLP-1 and appetite and energy intake [20]. Therefore, the main role of GLP-1 in pregnancy may not be to regulate appetite but to regulate other adaptations required for pregnancy; for example, GLP-1 is a key mediator of β-cell mass expansion in pregnancy, triggered in part by intra-islet GLP-1 release [21]. Studies of circulating incretin levels in pregnancies with GDM have provided inconsistent results. Although there is the potential for GLP-1 levels to be altered in pregnancies complicated by GDM due to increased glucose and fatty acids in the circulation, many studies have suggested that GLP-1 levels are lower in mothers with GDM [22] and continue to decline for longer into the post-partum period than they do in mothers without diabetes [19]. Contrary to these reports, a study in 2023 reported that maternal plasma GLP-1 was higher in pregnancies with LGA babies and positively correlated with birthweight [23]. Therefore, it is currently unclear whether GLP-1 levels are altered in pregnancies complicated by obesity or GDM, and if so in which direction. It is important to note that endogenous GLP-1 has a very short half-life, and activation of GLP-1 receptors by GLP1RAs that have a much longer half-life in circulation results in a different activation profile.

Functional L-cells have been reported in human fetuses from as early as nine-week gestation [24]. Therefore, the fetus may release excess GLP-1 in pregnancies where there is over-nutrition due to the maternal diet or GDM. Furthermore, a recent study in rats has shown that leptin is able to stimulate fetal intestinal cells to release GLP-1 [24]. As pregnancies complicated by obesity often have higher circulating leptin levels [25], this could also lead to a rise in endogenous fetal GLP-1. The GLP1R is expressed in the human and mouse placenta, and GLP1R activation by Exendin-4 stimulates PKA, ERK1/2 and mTOR pathways in primary human trophoblast cells in culture [23]. It is not yet known how this may influence nutrient transfer or fetal growth. Given the known sex-specific differences in placental function and fetal growth rate, it is also plausible that GLP-1RA effects on the placenta could vary by fetal sex.

Aside from the placenta, the GLP1R has been shown to be present in the fetal heart and brain, and neonatal lungs (Figure 2). These organs are all therefore potential sites of alerted signalling if endogenous fetal GLP-1 levels are altered, or if GLP-1 signalling is over-activated by exposure to GLP1RAs during fetal development. In adults, GLP1RAs increase mean heart rate via directly stimulating the sinoatrial node and enhancing sympathetic tone [26]. It is unknown whether there is a direct effect of GLP1RAs on the fetal heart with pregnancy use of GLP1Ras (in addition to a lack of knowledge regarding whether GLP1RAs enters the fetal circulation).

### Pre- pregnancy GLP1RA use and impact on maternal health

Although GLP1RA medications are not advised during pregnancy, use of GLP1RAs in the pre-pregnancy period has the potential for beneficial long-term effects by causing pre-pregnancy weight loss and allowing women to enter pregnancy metabolically healthier. However, as cessation of GLP1RAs is associated with a rapid increase in body weight due to weight rebound, concerns have been raised about the potential for excess gestational weight gain (GWG) in women who take the medication during the pre-pregnancy period and then stop taking it when they plan to conceive or fall pregnant.

Recent studies have conducted retrospective analysis of pregnancies where the mother was receiving GLP1RA medications in the perinatal period. A study by Imbroane et al. identified individuals who had filled a GLP1RA prescription within the 2 years preceding their pregnancy compared with a cohort matched for BMI and T2DM rates that had never been prescribed a GLP1RA [27]. The women who received GLP1RA medications in the two years prior to pregnancy were less likely to develop GDM and hypertensive disorders of pregnancy, and less likely to experience preterm delivery and undergo caesarean delivery. A similar study by Pondugula et al. identified patients who delivered a baby between 2014 and 2024 and had confirmed GLP-1RA medication in the one year preceding the pregnancy (and in some cases, there was a suggestion that the medication had been continued into early pregnancy) [28]. In this study, pre-pregnancy GLP-1RA medication was also associated with lower odds of hypertension in pregnancy when compared with unexposed individuals with pregestational diabetes and with unexposed individuals who were undergoing other weight management treatment. These associations were slightly more robust among the women who continued taking the medications during at least some of the pregnancy. Importantly, in this cohort, pre-pregnancy GLP-1RA use was also associated with increased gestational weight gain, which may reflect rebound weight gain in the women who ceased taking the drug when they became pregnant. Similarly, a case report of a woman who was taking semaglutide for PCOS (and had as a result lost 27 kg in weight while on the medication and entered pregnancy with a BMI of 29) ceased semaglutide at three weeks of gestation and went on to gain 35 kg in body weight over the course of the pregnancy [29]. This is three times over the IOM recommendation of 7–11 kg GWG for women in this BMI range. However, Guo et al. conducted a retrospective study of 42 women with obesity who had used GLP1RAs for six months pre-pregnancy, but with an eight-week withdrawal before pregnancy, compared with women with obesity who chose to manage their weight with diet and exercise, and found that women who had been using GLP1RAs pre-pregnancy had reduced GWG compared to the diet and exercise group [30]. The women using GLP1RA medication achieved greater weight loss in the pre-pregnancy period, so they entered pregnancy with a lower BMI compared with the women using diet and exercise. This was also associated with a reduced incidence of MASLD and improved lipid profile throughout pregnancy in those women with pre-pregnancy GP1RA use [30]. There are therefore no consistent data regarding stopping the medication pre-conception/early pregnancy is associated with better or worse pregnancy outcomes.

To date, four rodent studies have modelled pre-pregnancy administration of GLP1RAs and examined maternal metabolic health and pregnancy outcomes, with mixed findings (rodent studies summarised in Table 2). In one study in rats, liraglutide or semaglutide was given at a dose of 0.3 mg/kg daily via IP injection to obese female rats for four weeks then withdrawn one week before pregnancy [30]. Due to rapid rebound weight gain in this one-week period, the GLP1RA-treated rats entered pregnancy with no significant difference in body weight to obese animals treated with a vehicle in the pre-pregnancy period. The study reported no difference in GWG, but improved lipid profile throughout pregnancy in the GLP1RA-pre-treated dams, a lasting reduction in circulating leptin and adiponectin levels and correction of fasting hyperinsulinemia (the latter in semaglutide-treated dams only). This suggests that even if weight gain occurs after stopping GLP1RA medications before pregnancy, there could still be some lasting metabolic improvements. A separate study administered liraglutide at a dose of 0.3 mg/kg daily via subcutaneous injection to obese mice pre-pregnancy for four weeks, with drug administration again stopped one week before pregnancy [35]. This pre-pregnancy treatment group was compared with obese mice that were swapped onto a chow diet at the onset of pregnancy. Both liraglutide treatment and diet switch led to weight reduction and improved insulin sensitivity and enhanced fertility, particularly in the liraglutide group. Similar to human reports, the mice that received liraglutide in the pre-pregnancy period had significantly higher GWG compared with controls once the drug treatment was stopped. There was no improvement in glucose tolerance in late gestation in the mice that had received pre-pregnancy liraglutide compared with obese mice that had no GLP1RA intervention. Zhang et al. administered semaglutide at a dose of 30 nmol/kg daily via subcutaneous injection to obese mice for 22 days pre-pregnancy and observed a reduction in body weight and improvement of insulin sensitivity in the animals during the pre-pregnancy semaglutide treatment, which was associated with an improvement in fertility after the drug was stopped [36]. Finally, Wang et al. administered semaglutide at a dose of 0.3 mg/kg weekly via IP injection for four weeks and stopped one week before pregnancy [25]. The rats given semaglutide pre-pregnancy entered pregnancy lighter than obese animals given a vehicle injection and remained lighter than the vehicle-treated obese dams throughout pregnancy but had higher GWG than control dams on chow diet, suggesting weight rebound began to occur.

**Table 2:** Rodent studies of GLP1R agonist use before or during pregnancy and associated maternal or fetal outcomes

Study	Species	Drug	Dose	Timing of drug exposure	Maternal/ Fetal Outcome
Artunc- Ulkumen et al. [31]	Rat	Exenatide	10 µg/kg daily IP injection to STZ-induced diabetic females	4 weeks	Increased ovarian reserve and endometrial integrity in non-pregnant females.
Graham et al. [32]	Mouse	Exendin 4	10 µg/kg daily subcutaneous injection to lean females	1 week pre-pregnancy and throughout pregnancy	Decreased offspring birth weight, sex-specific alterations in neuronal pathways involved in anxiety in the offspring and increased body weight.
Rozo et al. [33]	Mouse	Exendin 4	1 nmol/kg daily subcutaneous injection to neonatal animals	Post-natal days 1–7	Rewiring of hypothalamic feeding circuitry, increased energy expenditure and reduced body weight in male and female neonates.
Younes et al. [34]	Rat	Liraglutide	0.3 mg/kg daily subcutaneous injection to rats with surgical hypertension	Pregnancy days 15–20	Lowered maternal blood pressure, no change in maternal body weight during pregnancy, reduced fetal growth in mixed-sex offspring.
Rodrigo et al. [35]	Mouse	Liraglutide	0.3 mg/kg daily subcutaneous injection to obese females	4 weeks pre-pregnancy then withdrawn 1 week before pregnancy	Improved insulin sensitivity before pregnancy and enhanced fertility. Increased GWG during pregnancy.
Guo et al. [30]	Rat	Liraglutide/ semaglutide	0.3 mg/kg daily IP injection to obese females	4 weeks pre-pregnancy then withdrawn 1 week before pregnancy	No change in GWG, improved lipid profile, reduction in circulating leptin and adiponectin, correction of fasting hyperinsulinemia (Sema-treated dams only) before and during pregnancy.
Wang et al. [25]	Rat	Semaglutide	0.3 mg/kg weekly IP injection to obese females	4 weeks pre-pregnancy then withdrawn 1 week before pregnancy	Decreased dam body weight before pregnancy, improved insulin sensitivity lasting into pregnancy. Increased GWG in Sema-treated dams compared with obese dams. No effect on male or female birth weight. Rescue of placental defects in male offspring from Sema-treated dams compared with obese dams.
Zhang et al. [36]	Mouse	Semaglutide	30 nmol/kg daily via subcutaneous injection to obese females	3 weeks pre-pregnancy then withdrawn at start of pregnancy	Decreased dam body weight and improved glucose homeostasis before pregnancy. Improved fertility. Improved male and female metabolic health in IVF offspring generated from Sema-treated dam oocytes compared with obese dam oocytes.
Qiao et al. [18]	Mouse	Semaglutide	6 µg/kg daily subcutaneous injection to lean females	Pregnancy days 13–17	No maternal weight loss during treatment in pregnancy, reduced fetal growth and altered placental structure in male and female fetuses.

Currently, the existing data from humans and rodents are therefore inconclusive as to whether pre-pregnancy treatment with GLP1RAs that is stopped before conception results in lasting improvements/detrimental effects to maternal health during pregnancy. It is not clear whether weight rebound consistently occurs after stopping GLP1RA agents, the time course of weight rebound, how it affects on body composition, and if this affects GWG in pregnancies complicated by obesity.

### GLP1RAs’ impact on fertility and reproduction

GLP1RAs have been increasingly recognised for their beneficial effects on fertility and reproductive function, particularly in women with polycystic ovary syndrome (PCOS). Prior to their widespread adoption for weight management, GLP-1RAs were primarily prescribed to women of reproductive age for PCOS, where they were shown to enhance menstrual cycle regularity and fertility outcomes [37,38]. While improvements in reproductive parameters have been largely attributed to weight loss and enhanced insulin sensitivity, emerging evidence suggests direct effects of GLP1RAs on the hypothalamic-pituitary–gonadal axis. There are several reports in the DHEA-induced PCOS mouse model that liraglutide and semaglutide decrease hyperinsulinemia and hyperandrogenemia and restore estrous cyclicity [39,40]. Independent of PCOS, preclinical studies indicate that GLP1R signalling may stimulate arcuate nucleus Kisspeptin neurons, contributing to reactivation of gonadotropin-releasing hormone (GnRH) secretion and restoration of luteinising hormone (LH) pulsatility [41]. In rodent models, both endogenous GLP-1 and GLP1RA treatments have been associated with increased preovulatory LH surges, enhanced follicular development and earlier onset of puberty [42]. Additionally, GLP1RAs reduce oxidative stress and inflammation in ovarian and endometrial tissues, with agents such as exenatide demonstrating protective effects on ovarian reserve and endometrial integrity in diabetic models [31]. Furthermore, GLP1RAs appear to promote vascularisation and mitigate against oxidative damage at the maternal–fetal interface, processes crucial for successful implantation and placental development [36]. Due to these direct effects on the hypothalamic–pituitary–gonadal axis, it is likely that GLP1RAs can alter fertility and reproductive function in women without PCOS or obesity, but studies assessing this in humans are lacking. The study by Imbroane et al. [27] matched women by their BMI, discounting any other health factors, and showed improvements in pregnancy outcomes after pre-conception use of any GLP1RA but did not examine fertility per se.

Collectively, these findings support a multifaceted role for GLP1RAs in improving reproductive outcomes beyond their metabolic effects, suggesting therapeutic potential in both fertility enhancement and potentially maternal–fetal health.

### Pregnancy GLP1RA use and impact on maternal health

Due to the fact that GLP1RAs are not currently licensed for use in pregnancy, there are no trials in humans examining what impact they have on the health of pregnant women. The official prescribing data for semaglutide advises only of potential harm to the fetus (see below), rather than to the mother in pregnancy. To our knowledge, there are no studies in animals that have reported maternal outcomes with GLP1RA use in pregnancy and compared with relevant control groups (in this case, animals who are obese and hyperglycemic in pregnancy). However, a study in rats showed that treatment with liraglutide before and during pregnancy significantly lowered maternal blood pressure and improved renal function in a model of hypertensive pregnancy without obesity [34].

### Pregnancy GLP1RA use and impact on fetal health

### Studies in rodents

The prescribing information from Novo Nordisk for semaglutide and from Eli Lilly for tirzepatide both advise against use in pregnancy due to adverse effects noted in animal models. In pregnant rats administered semaglutide at the human clinical dose for two weeks prior to mating and during pregnancy, there was increased fetal death and structural abnormalities. Specifically, Novo Nordisk reported reduced growth and fetuses with abnormal heart blood vessels and changes in skeletal structures (including cranial bones, vertebra and ribs) [43]. In rabbits and cynomolgus monkeys administered semaglutide during pregnancy, early pregnancy losses, structural abnormalities and reduced fetal weight were observed in fetuses at equivalent dose to clinical exposure (rabbit) and ≥2 fold the recommended human dose (monkey) [43]. In all cases, these findings coincided with maternal body weight loss due to reduction in food intake with semaglutide use. Recently, independent rodent models (Table 2) have also reported reduced fetal growth after administration of GLP1RAs to the mother in pregnancy [18,34]. While the study by Younes et al. using liraglutide reported the fetal growth was accompanied by maternal weight loss [34], a study by Qiao et al. using semaglutide reported fetal growth restriction, independent of maternal weight loss, and suggested the fetal growth restriction occurred due to effects on nutrient transporters in the placenta [18].

Given the known action of GLP1RAs in the brain, studies in rodents have examined the effects of GLP1RA exposure on the fetal and neonatal brain. Administration of the agent exendin 4 to dams during pregnancy results in sex-specific alterations in neuronal pathways involved in anxiety in the offspring and increased body weight [32], whereas post-natal administration of exendin 4 directly to neonatal mice resulted in rewiring of hypothalamic feeding circuitry and reduced body weight [33]. It is not yet known whether GLP1RAs administered to the mother can reach the fetal brain. However, if any of the GLP1RAs can cross the placenta, then they could directly influence fetal brain development, as exendin-4 has been shown to induce cell proliferation in vitro [44] and GLP1RAs administered subcutaneously to adult mice can promote neurogenesis in the brain [45] (hence the current interest in whether GLP1RAs could be used to treat neurodegenerative diseases). A recent study by Wang et al., which administered semaglutide only in the pre-pregnancy period to obese rats, reported that pre-pregnancy administration was sufficient to correct some of the changes to hypothalamic gene expression in fetal offspring [25], presumably via an improvement in maternal metabolic health.

### Studies in humans

In response to the increasing reports of GLP1RA exposure in pregnancy, several observational studies (summarised in Table 3) have been conducted screening large databases of pregnancy records to uncover the impact of perinatal GLP1RA exposure on fetal outcomes including congenital malformations and pregnancy loss. A study incorporating six centres that are members of the European Network of Teratology Information Services (ENTIS) found no increase in congenital anomalies or pregnancy loss, and no change in birth weight compared with control groups of women with diabetes and overweight/obesity in pregnancy [46]. In the group exposed to GLP-1RAs, the median gestational age at which the medication was stopped was five weeks, and most cases of exposure were reported by a healthcare provider to ENTIS. A similar observational population-based study combining data from four Nordic countries (in the period 2009–2020), the US MarketScan Database (from 2012 to 2021), and the Israeli Maccabi Health Services database (from 2009 to 2020) identified >900 cases where there had been a prescription fill for a GLP1RA from 90 days before pregnancy to end of first trimester [15]. Results from this study did not indicate a large increased risk of major congenital malformations in pregnancies with confirmed GLP1RA use above the risk conferred by maternal pre-existing T2DM alone. A retrospective review of an obstetric database at a tertiary obstetric hospital in Brisbane, Australia, identified 13 women who were exposed to semaglutide in the first trimester [47]. From these 13 pregnancies, one infant had significant cardiac anomalies in the context of poor maternal glycaemic control in the first trimester, as well as maternal obesity and hypertension throughout the pregnancy. No other major congenital malformations were observed. Most of the studies above, due to their study design, have captured women with either pre-pregnancy T2DM or GDM and compared women within these categories with/without GLP1RA use. A study combining data from clinics in Lebanon and the United States recently examined GLP1RA use in pregnant women without a history of T2DM or occurrence of GDM (so in most cases, the GLP1RA was being used for weight loss) and observed that babies born to mothers prescribed a GLP1RA in the 90 days prior to pregnancy were more likely to require a NICU stay than babies born to mothers not prescribed weight loss medications [48]. However, there was no increase in failure to thrive or congenital heart defects in the babies of mothers prescribed GLP1RAs in this study.

**Table 3:** Large-scale studies that have retrospectively examined the consequences of GLP1RA exposure in the perinatal period on fetal outcome

Study population	Inclusion criteria	Number of women with identified exposure	Study period	Control group	Outcome
European Network of Teratology Information Services [46]	Self or healthcare professional reported exposure in the first trimester	168	2009–2022	Women with obesity or GDM	No increase in congenital abnormalities or pregnancy loss and no change in birth weight
Four Nordic countries, US MarketScan Database and Israeli Maccabi Health Services database [15]	Prescription fill for a GLP1RA from 90 days before pregnancy to end of first trimester	900	2009–2020	Women with T2DM	No increase in congenital abnormalities
Maternity database in Brisbane, Australia [47]	Self or healthcare professional reported use of GLP1RA in the first trimester	13	2021–2023	Healthy pregnant women	One infant had significant cardiac anomalies
Cosmos, a dataset representing more than 256 million patient records from all 50 states and Lebanon [48]	Prescribed a GLP1RA in the 90 days prior to pregnancy	1163	2017–2024	Healthy pregnant women	Increased percentage of NICU admissions of neonates
US FDA Adverse Event Reporting System database [49]	Self or healthcare professional reported exposure in pregnancy	354	2004–2023	Women with obesity	Adverse events reported in reproductive category, including spontaneous abortion and pre-eclampsia

These studies have collected data after any GLP1RA exposure (exenatide, dulaglutide, liraglutide, semaglutide and tirzepatide). Due to the timing of the data collection, most studies do not include cases of exposure to tirzepatide.

The most concerning study examining GLP1RA exposure in human pregnancy is a recent screen of the US FDA Adverse Event Reporting System database from 2004 to 2023 [49]. This study reported that the most common reasons for use of GLP1RAs in women who became pregnant while taking them were type 2 diabetes (51.41%), weight control (17.51%) and polycystic ovaries (1.69%), and the most frequently used GLP1RAs in these women were liraglutide (42.37%), semaglutide (29.94%) and dulaglutide (14.69%). The database search identified 1671 adverse drug reactions reports involving GLP1RA use in pregnancy in the 20 year period, with an exponential rise in reported cases since 2012. There were significant adverse drug reaction signals in the reproductive and gastrointestinal categories, with spontaneous abortion and pre-eclampsia being the most concerning. The data showed a heightened risk for adverse events with GLP1RA use in pregnancy across all age groups except for those aged 20–24 years. The high frequency of cases and detailed prescribing information that was available in most cases allowed the study authors to demonstrate a dose-dependent association with increased adverse events for liraglutide.

There are an increasing number of case studies in the literature reporting on individual cases of exposure to GLP1RAs during pregnancy (summarised in Table 4). The majority of these reports are in women who were taking a GLP1RA agent for treatment of T2DM and stopped the medication during the first trimester (although in one instance the medication was continued through to term of pregnancy). In the majority of cases, the pregnancy resulted in a healthy baby, although in one case, the baby was born with an atrial defect that spontaneously resolved by age three [56], and one baby had mild bilateral renal pyelectasis [53]. One baby was born LGA (in the absence of GDM in the mother, but in a case of high GWG) resulting in shoulder dystocia at birth [29]. These case studies also highlight that many women were taking GLP1RAs off-license or through online purchase, meaning that the actual numbers of pregnancies with exposure in the first trimester could be a lot higher than screening of databases based on prescription by clinicians suggests.

**Table 4:** Human case studies of GLP1RA use in individual pregnancies and associated maternal and fetal outcomes

Study	Drug	Maternal condition	Last confirmed maternal dose (week of gestation)	Fetal outcome	Maternal outcome
Greco et al. [50]	Liraglutide	T2DM	13	Healthy newborn	
Ivanisevic et al. [51]	Liraglutide	T2DM	Term	Healthy newborn, healthy at two years of age	
Burlina et al. [52]	Dulaglutide	T2DM	15	Neonatal jaundice but otherwise healthy	
Alghamdi et al. [53]	Dulaglutide	T2DM	13	Healthy newborn	
Williams et al. [54]	Exenatide	T2DM	14	Healthy newborn	Hypertension followed by preeclampsia
Skov et al. [29]	Semaglutide	PCOS	3	LGA but otherwise healthy newborn, healthy at 6 months of age	High GWG resulting in shoulder dystocia
Molteni et al. [55]	Dulaglutide	T2DM	33	Healthy newborn	Polyhydramnios
Dogan et al. [56]	Liraglutide	T2DM	Pregnancy 1: 17	Newborn had atrial septal defect that corrected by age 3, healthy at 7 years of age	
			Pregnancy 2: 13	Healthy newborn, healthy at 5 years of age	

## Remaining questions and priorities for future research

### Maternal vs. offspring effects

**Figure 3:**
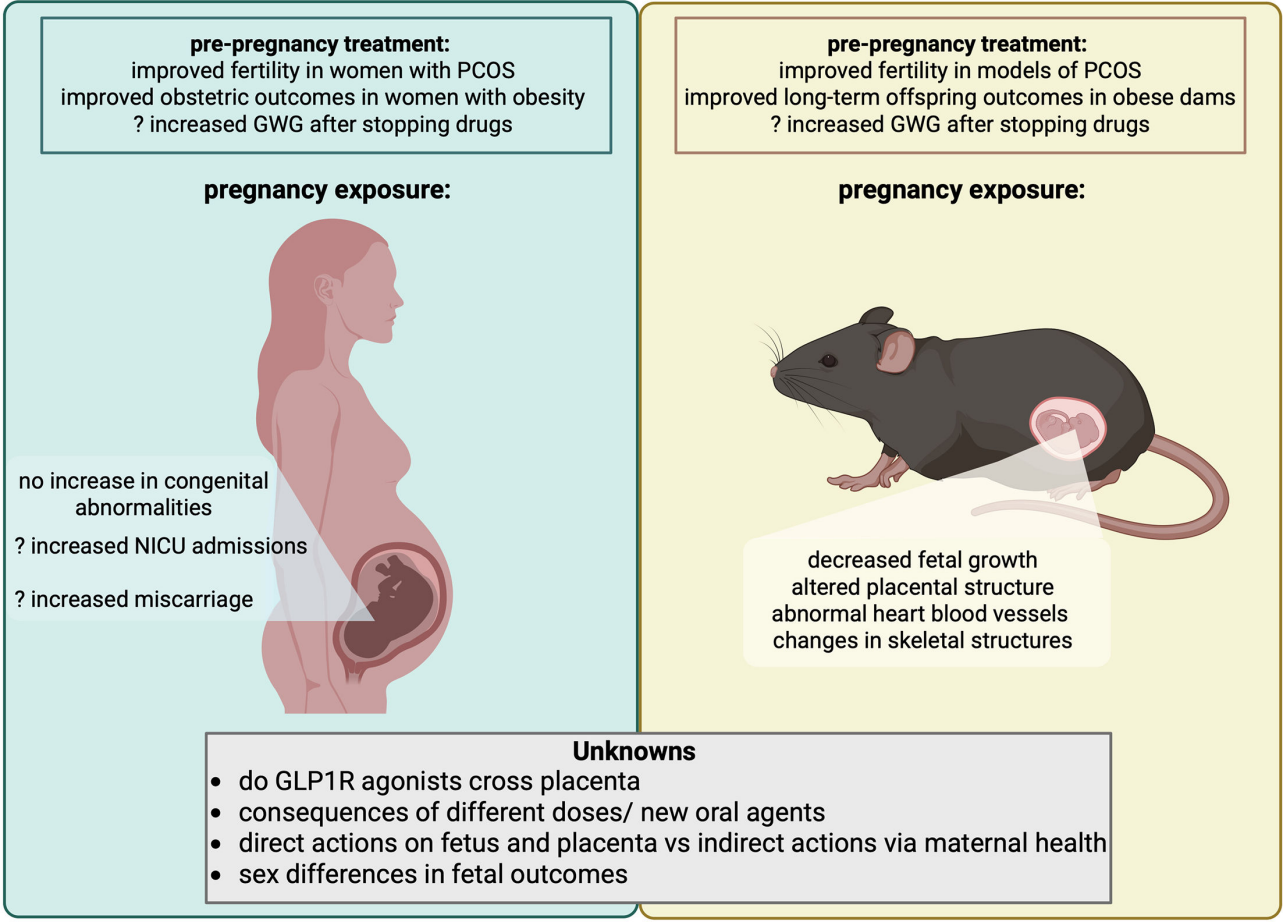
Summary of human and rodent models of pre-conception or pregnancy use of GLP-1 receptor agonists. A summary of results from human and rodent studies examining GLP-1 receptor agonist use in the mother exclusively pre-pregnancy, or during pregnancy. Human and rodent studies have reported that GLP1R agonists used pre- pregnancy improve fertility in PCOS; rodent studies suggest this is via a direct impact on the hypothalamic- pituitary- gonad axis in addition to the improvement in maternal metabolic health. There are conflicting reports within both human and rodent studies whether there is increased gestational weight gain during pregnancy after GLP1R agonist use is stopped at conception. In humans, GLP1R agonist exposure in pregnancy has not been linked with increased congenital abnormalities, but there are reports of increased risk of miscarriage and NICU admission after birth. In rodents, GLP1R agonist exposure in pregnancy is associated with decreased fetal growth, altered placental structure, abnormal heart structure and changes to the fetal skeleton.

The limited data from preclinical and human studies on GLP1RA use during pregnancy suggest a complex interplay between effects on maternal and offspring health, and there are currently no data at all about short-term (i.e. on the mother and labour outcomes) versus long-term (i.e. on future health of the mother and child) outcomes. Potential benefits or harms may affect maternal and fetal systems differently at different stages of pregnancy, much like what is observed with metformin use during pregnancy [57]. Despite this, given the current limitations in treatment options for GDM and the absence of approved therapies for obesity during pregnancy, the use of GLP1RAs during specific time windows periconceptionally or at specific doses warrants serious consideration (Figure 3).

### Can GLP1RAs cross the placenta?

Endogenous GLP-1 does not cross the placenta, likely due to its rapid degradation by DPP4 that is expressed by the placenta. However, synthetic GLP-1RAs, such as semaglutide, are designed with DPP4-resistant modifications to extend their half-life. However, these compounds are large molecules – liraglutide (~3700 g/mol) and semaglutide (~4100 g/mol) – and drugs of this size typically do not cross the placenta unless active transport mechanisms exist. Nevertheless, placental transfer can differ in pregnancies complicated by obesity or systemic inflammation. In a mouse study [58], fluorescently labelled exendin 4 injected to the dam was not detected in the fetus or placenta under normal conditions. However, in obese mice with systemic inflammation, the compound was observed in the fetal membranes, suggesting altered barrier permeability with obesity. This is an interesting parallel to the access of GLP1RAs to the brain. In adult animals, GLP1RAs reach the brain via circumventricular organs, which lack a fully formed blood–brain barrier (BBB). Not only is the fetal BBB immature during early brain development, but BBB function is disrupted in fetal and neonatal offspring of obese mothers, meaning there could be increased fetal hypothalamic exposure to GLP1RAs in the population of women who would be taking GLP1RAs during the perinatal period.

### Are fetal effects independent of maternal weight loss?

Fetal outcomes may vary depending on the mother’s metabolic status and the degree of weight loss induced by GLP1RA treatment. Substantial maternal weight loss during pregnancy could contribute to fetal growth restriction. Conversely, if the treatment results in limited weight loss and persistent hyperglycemia or hyperlipidemia, elevated fetal exposure to endogenous GLP-1 could theoretically promote growth. This may explain the different results seen in studies correlating endogenous circulating GLP-1 to fetal growth, compared with models of GLP1RA use in pregnancy. Animal models, including pair-fed control groups, will be essential to understand whether any fetal outcomes are due to maternal weight loss.

There is currently limited understanding of whether the central effects of GLP-1 in pregnancy mirror those in the non-pregnant state. A recent study in mice showed that vagal afferents are desensitised during pregnancy [59], which may alter the ability of GLP1RAs to act on vagal pathways. Thus, it is unclear whether these medications would produce the same appetite-suppressing or weight-reducing effects during pregnancy as they do outside it – a major concern when considering their use during gestation.

### Newer incretin-based therapies and considerations for pregnancy

Emerging therapies that target multiple incretin pathways – such as Retatrutide (a triple agonist targeting GLP-1, GIP and glucagon receptors) and Cagrisema (a dual agonist mimicking GLP-1 and amylin) – will require thorough investigation for their safety and efficacy in pregnancy. Long-acting formulations, such as Maritide (a GLP-1 agonist and GIP antagonist administered monthly), may offer lower fetal exposure, depending on timing. However, longer half-lives of newer drugs raise concerns about residual drug exposure even if treatment is stopped as soon as pregnancy is detected and might require a longer washout period.

### Limiting inter-generational transmission of obesity risk

GLP-1RAs may offer a promising strategy for interrupting the intergenerational transmission of obesity and metabolic disease. In a study by Zhang et al., semaglutide administered to obese female mice prior to pregnancy improved the metabolic health of their offspring compared with obese controls [36]. These benefits suggest the potential to reduce future obesity and GDM risk in female offspring, breaking the intergenerational transmission of obesity risk. Furthermore, improving glycemic control and reducing weight gain during pregnancy will have positive implications for future pregnancies as they will allow mothers to enter subsequent pregnancies in a healthier state.

In conclusion, the advent of incretin-based therapies has been game-changing in the context of weight loss medications and our ability to treat one of the major health care issues of the 21st-century obesity. However, their potential use in the context of gestational diabetes and obesity during pregnancy and whether they represent a potential means by which the transmission of poor cardiometabolic health from mother to child can be halted remains to be established and awaits the results of ongoing studies that will answer key knowledge gaps (Figure 3).

## Open Access

This article has been published open access under our Subscribe to Open programme, made possible through the support of our subscribing institutions, learn more here: https://portlandpress.com/pages/open_access_options_and_prices#conditional

## Abbreviations

BBB: Blood Brain Barrier
GDM: gestational diabetes mellitus
GLP-1: glucagon-like peptide-1
GLP1RAs: GLP-1 receptor agonists
GWG: Gestational Weight Gain
IP: Intra-Peritoneal
LGA: large for gestational age
NICU: Neonatal Intensive Care Unit
PCOS: Polycystic Ovary Syndrome
T2DM: Type 2 Diabetes Mellitus
